# Supplementary material for: Rv0687 a Putative Short-Chain Dehydrogenase Is Required for In Vitro and In Vivo Survival of Mycobacterium tuberculosis
Source: Int J Mol Sci. 2024 Jul 18;25(14):7862. doi: 10.3390/ijms25147862 (PMC11277061; doi:10.3390/ijms25147862)
Supplement: Supplementary file 1 [file ijms-25-07862-s001.zip › ijms-3078526-supplementary.pdf]

Construction and Confirmation of RvΔ0687 and its complemented strain

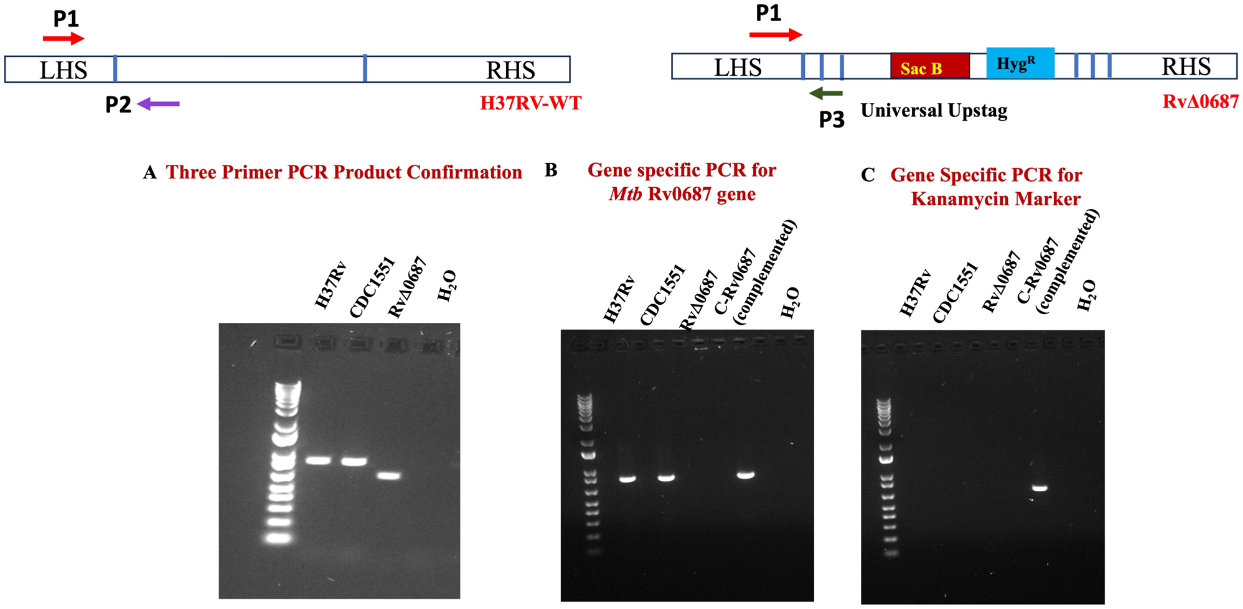

Figure S1. Construction of gene deletion mutant of Rv0687 and its complementation: Rv0687 was deleted in *Mtb* using specialized transduction and confirmed by three primer PCR A-C. **(A)** Three primer PCR product confirmation **(B)** Gene specific PCR for *Mtb* Rv0687 gene **(C)** PCR for Kanamycin marker to confirm the complemented strain. This was performed using H37Rv, CDC1551, RvΔ0687 and C-Rv0687 strains.

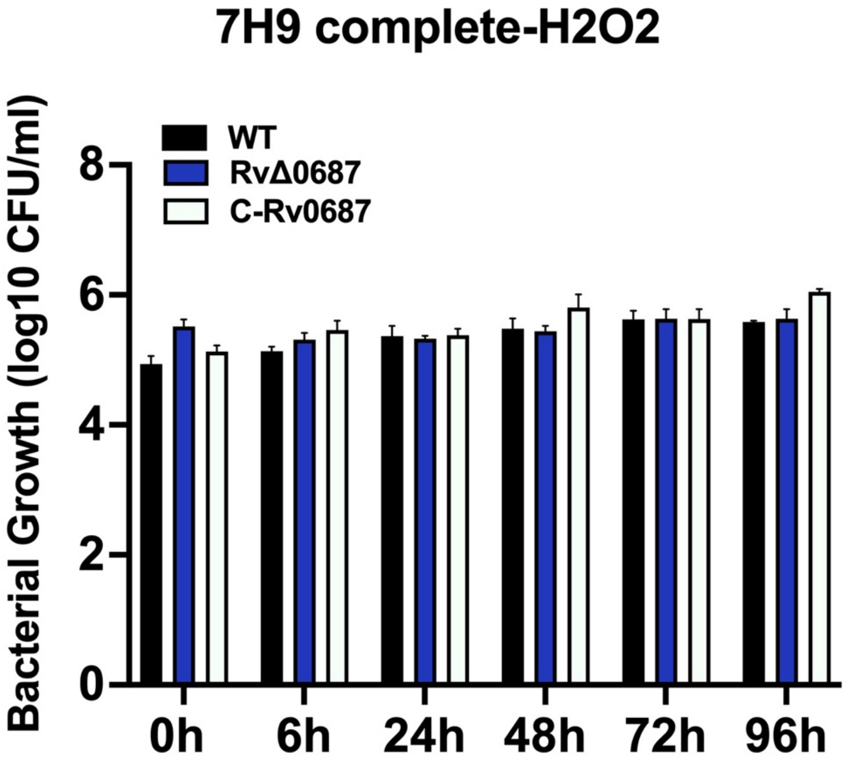

Figure. S2 Effect of H_2O_2 in 7H9 complete media. The WT, RvΔ0687 and C-Rv0687 strains were grown in 7H9 complete media and exposed to 5mM H2O2 and all the bacterial strains were monitored for growth at 0, 6, 24, 48, 72 and 96h and CFUs were estimated after 3 weeks of incubation and calculated for per ml of bacterial culture

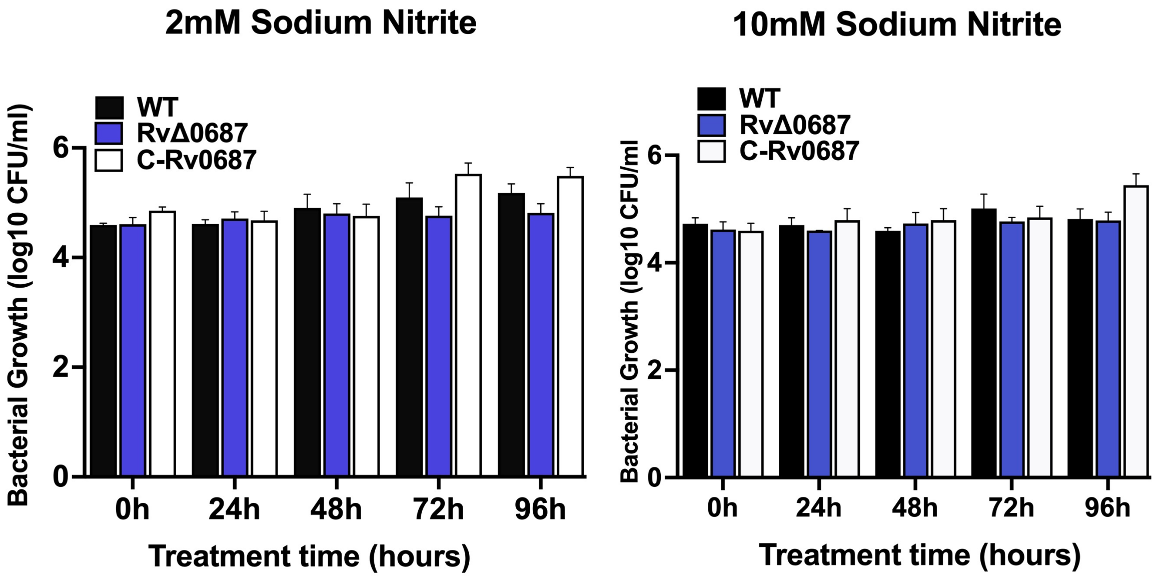

Figure S3. Effect of Sodium Nitrite Stress: The WT, RvΔ0687 and C-Rv0687 strains were grown in 7H9 dextrose media and exposed to 2 or 10mM sodium nitrite and all the bacterial strains were monitored for growth at 0, 24, 48, 72 and 96h and CFUs were estimated after 3 weeks of incubation and calculated for per ml of bacterial culture.

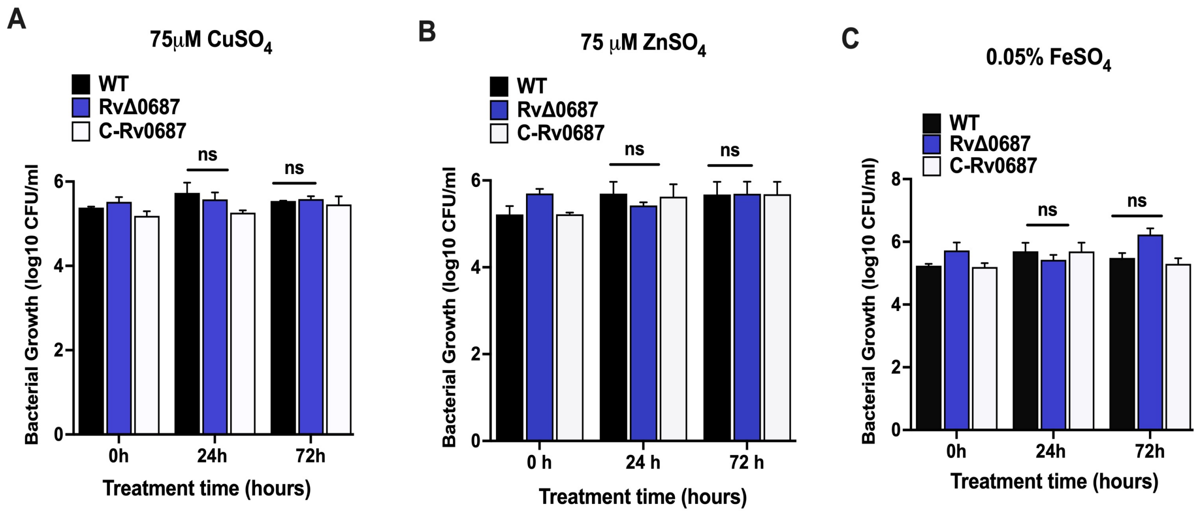

Figure S4: Response of WT, RvΔ0687 and C-Rv0687 to Metal Stress: The WT, RvΔ0687 and C-Rv0687 strains were grown in 7H9 dextrose media and exposed to (A) 75μM $CuSO_4$, (B) 75μM $ZnSO_4$ or (C) 0.05% $FeSO_4$ and the cultures were monitored for growth at 0, 24 and 72h. The CFUs were analyzed by serially diluting the cultures and plating on 7H10 plates and CFUs were calculated per ml of bacterial culture.

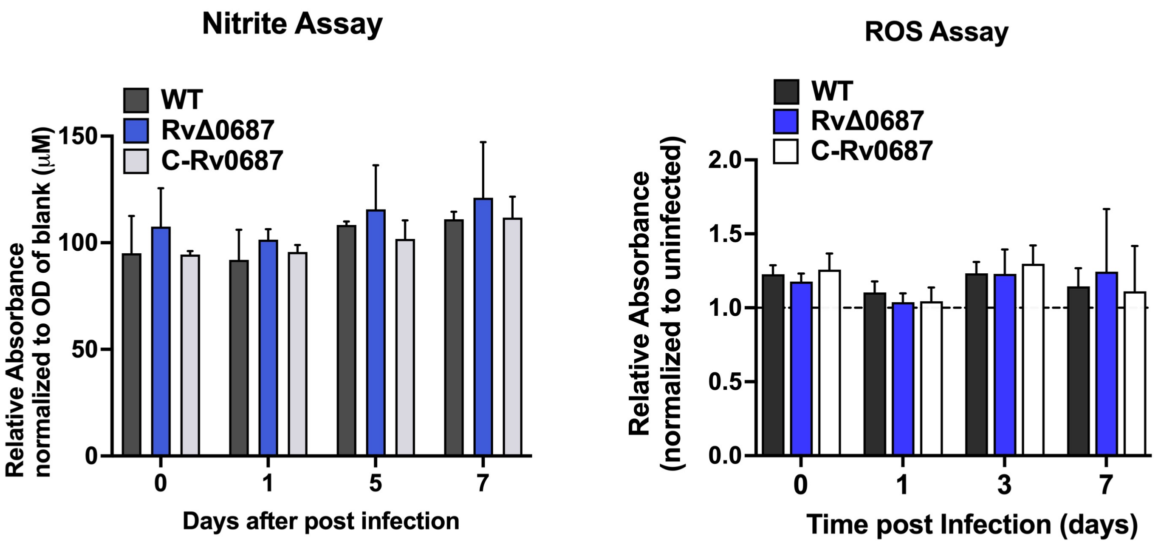

A B

Figure. S5 A-B. RNS and ROS production by BMDMs in response to infection

(A) Assessing the RNS production by BMDMs during Infection: The RNS production was measured in the cell free supernatants of BMDMs infected with WT, RvΔ0687 or C-Rv0687 strains at Day 0, 1, 5 and 7 (**B) Assessing the ROS production by BMDMs during Infection:** The production of ROS was measured at Day 0, 1, 3 and 7 in the post infected lysates of BMDM infected with WT, RvΔ0687 or C-Rv0687 strains using optical density readings, using uninfected lysates as a control.

Table S1: List of strains used in the current study. Abbreviations used *hyg*: hygromycin casette, *sacB*: counter selectable marker.

Strain No	Strain name	Genotype	Description	Source/Reference
CDC1551	WT	wild type		Colorado state University
ST1	RvΔ*0687*	RvΔ0687::*hyg sacB*	Specialized transduction of CDC1551 with phasmid phST1	This study
ST2	C-Rv0687	RvΔ0687::*hyg sacB*::P_{hsp60} Rv0687	Transformation of ST1 with plasmid pST2	This study

Table S2: List of plasmids and phasmids used in the current study: Abbreviations used *hyg*^{R}: hygromycin resistance, *suc*^{S}: Sucrose sensitive, *kan*$^{R:}$ Kanamycin resistance.

Plasmids/phages	Description	Reference
P004s	Suicide recombination delivery vector carrying *hyg*^{R} *sacB* for gene disruption, *hyg*^{R}	Jain et., al 2004
pST1	P004s containing homologous left and right arm to delete Rv0687, *hyg*^{R}, *suc*^{S}	This study
phAE159	Conditionally replicating shuttle phasmid vector	Jain et., al 2004
phST1	Phsmid with flanking regions to delete Rv0687 by homologous recombination, *hyg*^{R}, *suc*^{S}	This study
pMV361	Integrative mycobacterial shuttle vector with hsp60 promoter carrying *kan*^{R} resistant gene	Strover et., al 1991
pST2	Rv0687 was cloned in pMV361, *kan*^{R}	This study

Table S3: Primers used in the current study. Abbreviations used AES*:* Allelic exchange substrate KO- knockout deletion strain.

Name of Primer	Sequence	Purpose
Rv0687_ LL	TTTTTTTTGCATAAATTGCTCACGGCTCGAAAACTGGTG	to construct AES
Rv0687_ LR	TTTTTTTTGCATTTCTTGCAGTCTGGCGTCACGTCACTTGATGT CTCACTGAGGTCTCTGGCCGTGCAGTGATCCTC	to construct AES
Rv0687_ RL	TTTTTTTTCCATAGATTGGTTGAATGCTTTGACTGGCGCCGAG TGTCTGGTCTCGTAGCACTGTCGGGCAATCAGATCC	to construct AES
Rv0687_ RR	TTTTTTTTCCATCTTTTGGGGCTCAACCAGCACCACATC	to construct AES
Rv0687_ L	GTCATCGCCAGTGTGACC	to confirm KO
Rv0687_ R	GTCGCGAATATCCACCTC	to confirm KO
Univers al uptag	ATGTCTCACTGAGGTCTCT	to confirm KO

Rv0687 Forward	CCCCAGCTGGTGTCTGCGCGGGGAGGATC	Complementation and to confirm complementation
Rv0687 Reverse	CCCAAGCTTAATACTTCAAGGCACCCTTA	Complementation and to confirm complementation
Kan_F	CTCGAGGCCGCGATTAAATTC	To confirm complementation
Kan_R	AAACTCACCGAGGCAGTTC	To confirm complementation
